# Mitochondrial genomic characteristics and phylogenetic analysis of a brewing fungus, *Rhizopus microsporus* Tiegh. 1875 (Mucorales: Rhizopodaceae)

**DOI:** 10.1080/23802359.2024.2356133

**Published:** 2024-05-20

**Authors:** Yue Deng, Guangjiu Chen, Xuedong Bao, Jie He, Qiang Li

**Affiliations:** a Luzhou Vocational and Technical College, Luzhou, Sichuan, P. R. China; b School of Food and Biological Engineering, Chengdu University, Chengdu, Sichuan, P. R. China

**Keywords:** Mitochondrial genome, brewing fungus, evolution, phylogeny

## Abstract

*Rhizopus microsporus* Tiegh. 1875 is widely used in a variety of industries, such as brewing, wine making, baking, and medicine production, as it has the capability to break down proteins and generate surface-active agents. To date, the mitochondrial genome features of early evolved fungi from the *Rhizopus* genus have not been extensively studied. Our research obtained a full mitochondrial genome of *R. microsporus* species, which was 43,837 bp in size and had a GC content of 24.93%. This genome contained 14 core protein-coding genes, 3 independent ORFs, 7 intronic ORFs, 24 tRNAs, and 2 rRNA genes. Through the use of the BI phylogenetic inference method, we were able to create phylogenetic trees for 25 early differentiation fungi which strongly supported the major clades; this indicated that *R. microsporus* is most closely related to *Rhizopus oryzae*.

## 1. Introduction

*Rhizopus microsporus* Tiegh. 1875 is a soil-borne filamentous fungus with a high ability to degrade protein and produce surface-active agents (Jennessen et al. 2005; de Barros Ranke et al. 2020). It has been isolated from various habitats including soil, decaying matter, and clinical samples (Yao et al. 2021). This species has been found to have a high degree of adaptability and is able to grow under a wide range of environmental conditions (Zhang et al. 2015; Yuwa-Amornpitak and Chookietwatana 2018). *R. microsporus* has diverse industrial applications. It is commonly used in brewing, wine making, baking, and medicine production due to its ability to degrade protein and produce surface-active agents (Celestino et al. 2006; Martínez-Ruiz et al. 2018). Surface-active agents produced by *R. microsporus* have been found to have biocontrol activities against plant diseases and act as biocides against bacteria and fungi (Orikasa et al. 2018; Škríba et al. 2020; Xiang et al. 2021). These properties make *R. microsporus* a highly valuable resource for industrial and agricultural applications.

Eukaryotes have a mitochondrial genome, which is indispensable in the regulation of growth and development, sustaining the cell’s homeostasis and enabling it to react to the environment (Ernster and Schatz 1981; McBride et al. 2006; Murphy 2009). It is suggested that the mitochondrial genome is a useful resource for examining fungal phylogeny (Xu and Wang 2015; Li, Bao et al. 2022, Li, Li et al., 2022; Li et al., 2023). To date, the mitochondrial genome characteristics of early differentiated fungi from the *Rhizopus* genus have been not well understood, with only two fungal mitochondrial genomes from the genus reported (Liang et al. 2022). In this study, we first obtained the complete mitochondrial genome of *R. microsporus*, which promotes understanding of the genomic characteristics of early differentiated fungi

## 2. Materials and methods

### 2.1. Sample collection

**Figure 1.**
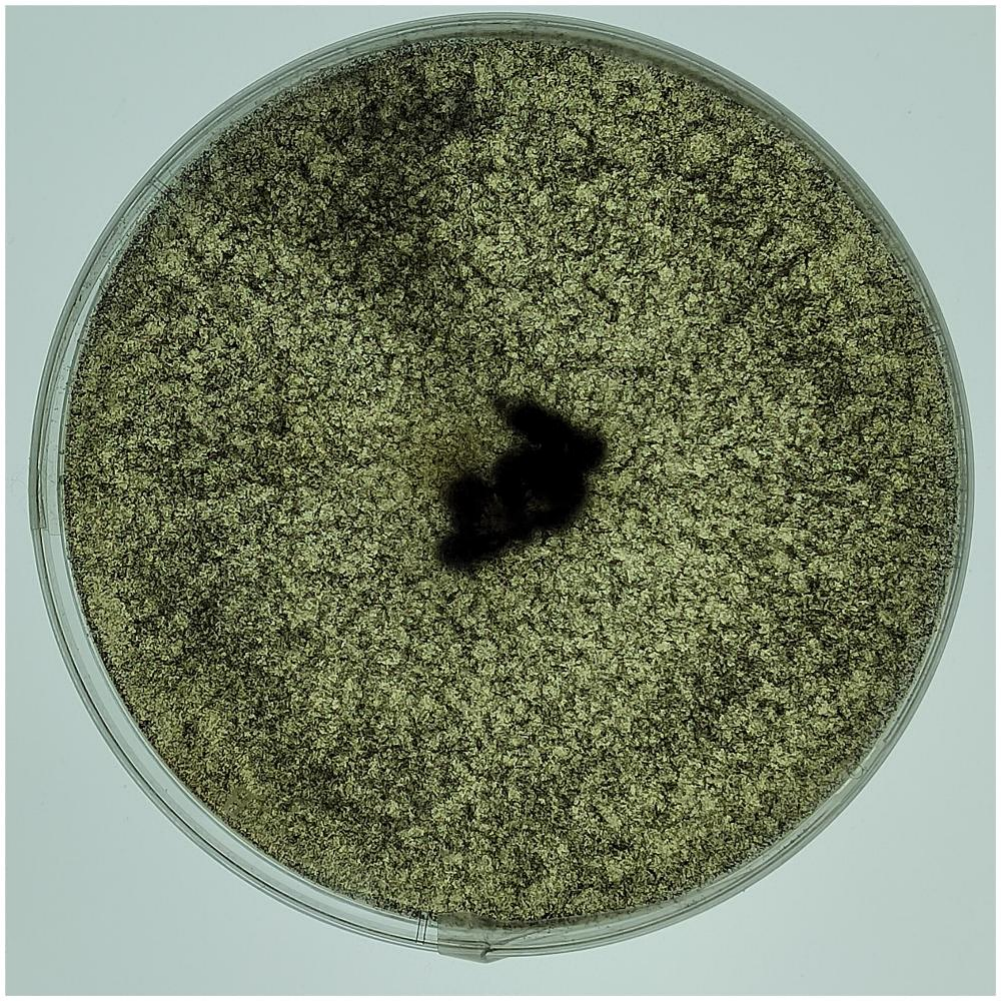
Isolation of the brewing fungus *Rhizopus microspores*. Photo of the species was taken by Qiang Li.

In 2023, a specimen of *R. microsporus* was isolated from a wine fermentation system in Luzhou (E 105.40°, N 28.91°), Sichuan, China. Morphological and ITS rDNA sequencing were used to identify the specimen, which was then deposited at Culture Collection Center of Chengdu University (contact person: Qiang Li; email: leeq110@126.com) with the voucher number Rmic1 (Figure 1).

### 2.2. Mitochondrial genome assembly and annotation

For DNA extraction of *R. microsporus*, a fungal DNA extraction kit from Omega Bio-Tek (Norcross, GA, USA) was utilized. The NEBNext® Ultra™ II DNA Library Prep Kit (NEB, Beijing, China) was then employed for sequencing library preparation as per the manufacturer’s instructions. Subsequently, the Illumina HiSeq 2500 Platform (Illumina, San Diego, CA, USA) was used for whole genome sequencing. To guarantee the accuracy of the data, ngsShoRT (Chen et al. 2014) was used to filter out low-quality sequences and AdapterRemoval v2 (Schubert et al. 2016) was employed to remove adapter reads. The mitochondrial genome of *R. microsporus* was de novo assembled using the version 4.3.3 of NOVOPlasty, with a k-mer size of 31 (Dierckxsens et al. 2017). The mitochondrial genome was annotated in accordance with our previously described methods (Li et al. 2019, 2020, 2023), which involved the use of the MFannot tool (Valach et al. 2014) and MITOS (Bernt et al. 2013). By using the NCBI Open Reading Frame Finder, we can forecast or modify PCGs or ORFs that are longer than 100 amino acids (Wu et al. 2017). Annotation of the functions of PCGs or ORFs was accomplished through BLASTP searches against the NCBI non-redundant protein sequence database (Bleasby and Wootton 1990). Exon and intron boundaries of PCGs were accurately identified with the help of exonerate version 2.2 (Slater and Birney 2005). Through the application of tRNAscan-SE v1.3.1, we ascertained and confirmed the presence of tRNA genes in the *R. microsporus* mitochondrial genome (Lowe and Chan 2016). OGDraw v1.2 was employed to generate a graphical representation of the mitochondrial genome (Lohse et al. 2013). The structures of intron-containing genes were visualized using the PMGmap online web (http://www.1kmpg.cn/pmgmap) (Zhang et al. 2024).

### 2.3. Phylogenetic analysis

The phylogenetic tree was built using methods that had been described previously (Li et al. 2020, 2021, 2022). Utilizing the MAFFT v7.037 software, we initiated the process by aligning individual mitochondrial genes (excluding intron regions) (Katoh et al. 2019). Utilizing SequenceMatrix v1.7.8, we connected the aligned mitochondrial genes to form a single, unified mitochondrial dataset (Vaidya et al. 2011). In order to detect any phylogenetic discrepancies between distinct mitochondrial genes, an initial partition homogeneity test was performed using PAUP v 4.0b10 (Swofford 2002) according to previous studies (Xiang et al. 2013). PartitionFinder 2.1.1 was utilized to pinpoint the most suitable models of partitioning and evolutionary processes for the merged mitochondrial dataset (Lanfear et al. 2017). MrBayes v3.2.6 was utilized to construct phylogenetic trees by applying Bayesian inference (Ronquist et al. 2012).

## 3. Results

**Figure 2.**
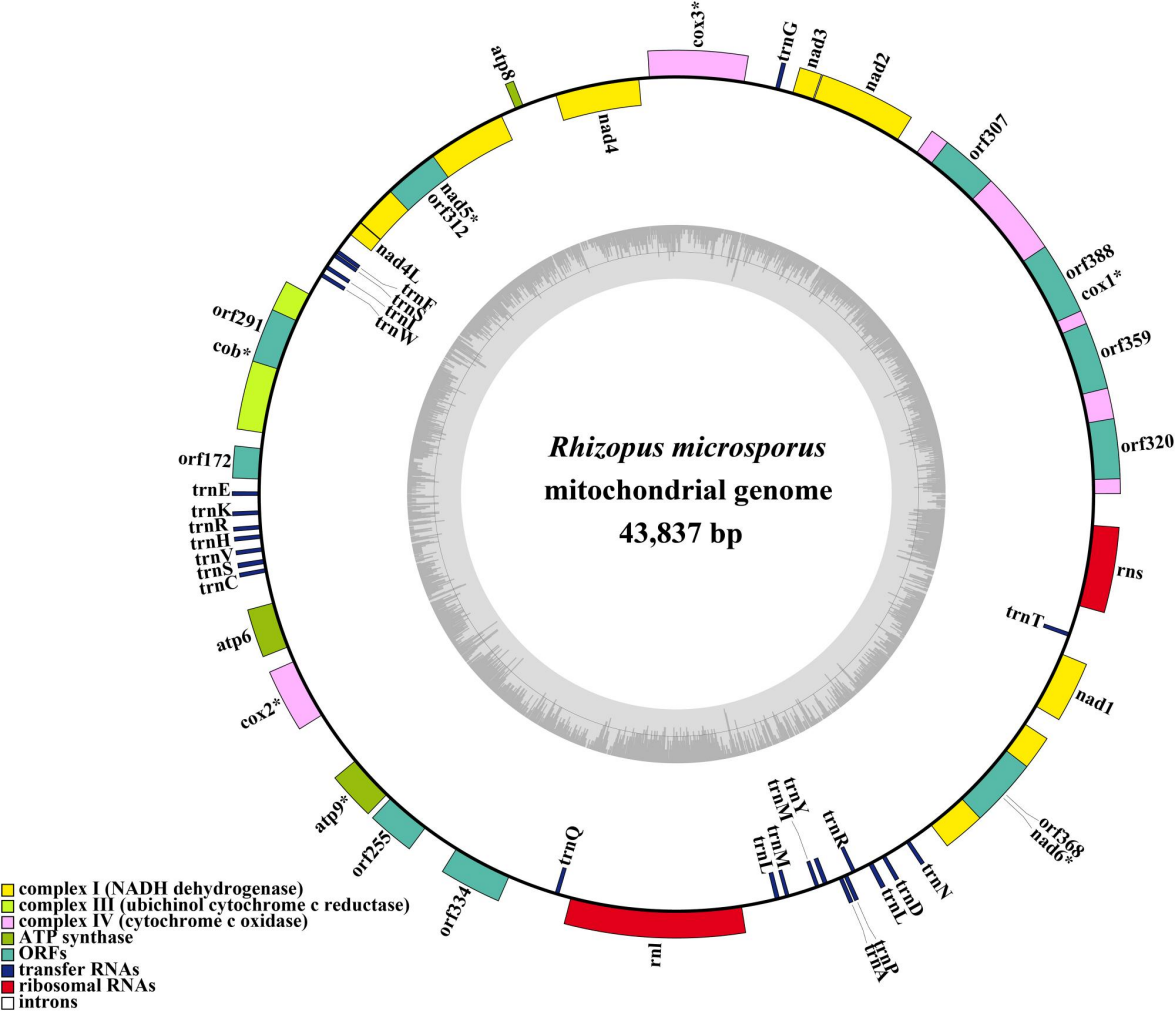
The Circular mitochondrial genome map of *Rhizopus microspores.*

**Figure 3.**
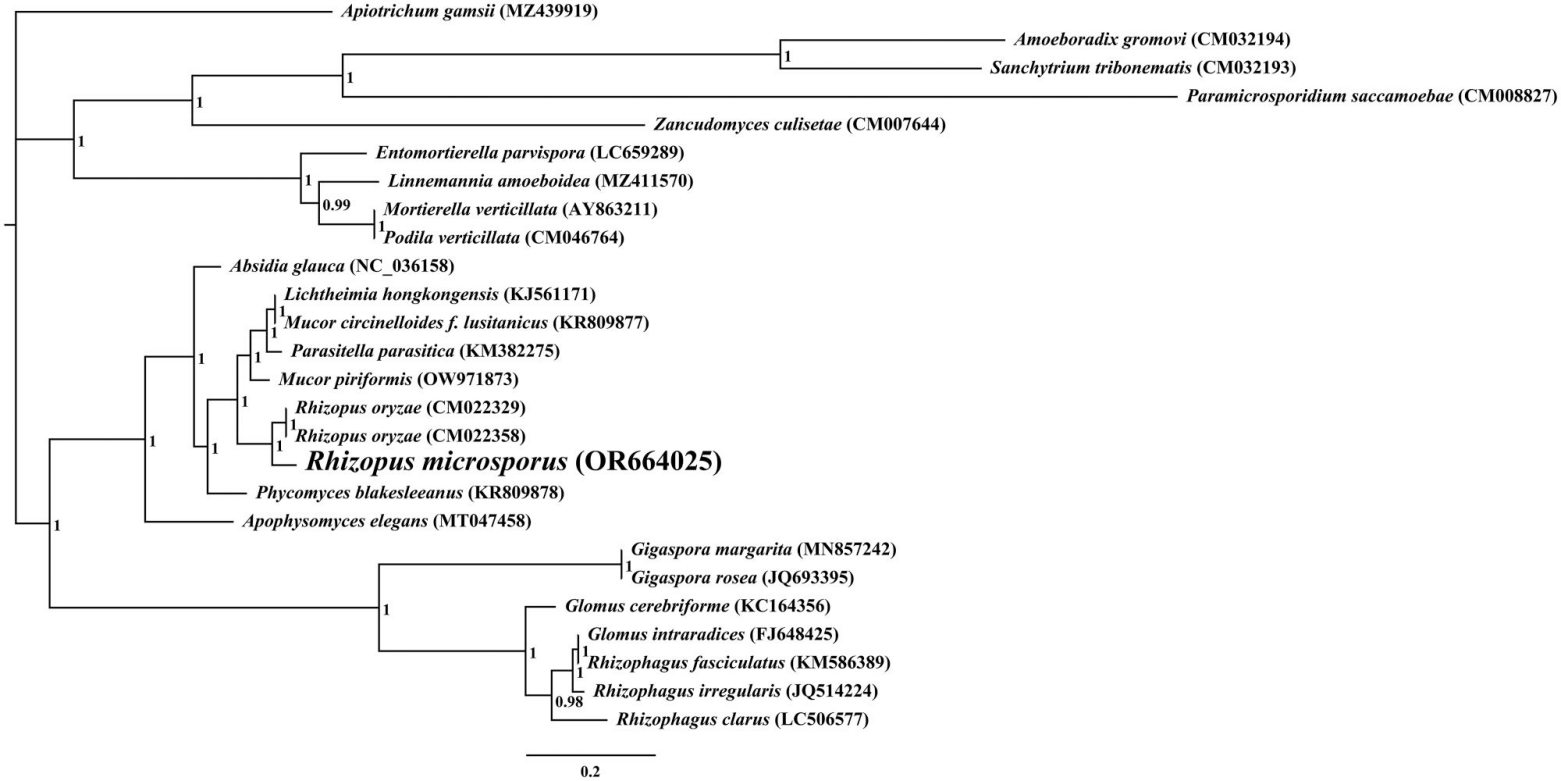
Bayesian inference (BI) tree generated using 14 concatenated mitochondrial protein-coding genes (*atp6*, *atp8*, *atp9, cob, cox1, cox2, cox3, nad1, nad2, nad3, nad4, nad4L, nad5,* and *nad6*) from *rhizopus microspores* and 25 other fungal species. *Apiotrichum gamsii* was set as the outgroup (Li et al. 2023). the accession number information of the sequence is as follows: *Rhizophagus clarus* (LC506577) (Kobayashi et al. 2018), *Rhizopus microsporus* (OR664025), *podila verticillata* (CM046764) (Morales et al. 2022), *lichtheimia hongkongensis* (KJ561171) (Leung et al. 2014), *rhizophagus irregularis* (JQ514224) (Formey et al. 2012), *gigaspora rosea* (JQ693395) (Nadimi et al. 2012), *linnemannia amoeboidea* (MZ411570) (Yang et al. 2022), *zancudomyces culisetae* (CM007644), *Mucor circinelloides f. lusitanicus* (KR809877), *amoeboradix gromovi* (CM032194) (Galindo et al. 2021), *glomus cerebriforme* (KC164356) (Beaudet et al. 2013), *parasitella parasitica* (KM382275) (Ellenberger et al. 2014), *sanchytrium tribonematis* (CM032193) (Galindo et al. 2021), *mortierella verticillata* (AY863211) (Seif et al. 2005), *entomortierella parvispora* (LC659289) (Herlambang et al. 2022), *absidia glauca* (NC_036158) (Ellenberger et al. 2016), *gigaspora margarita* (MN857242) (Venice et al. 2020), *apophysomyces elegans* (MT047458), *paramicrosporidium saccamoebae* (CM008827) (Quandt et al. 2017), *mucor piriformis* (OW971873) (Papp et al. 1999), *rhizophagus fasciculatus* (KM586389) (Wang et al. 2020), *phycomyces blakesleeanus* (KR809878), *rhizopus oryzae* (CM022329) (Seif et al. 2005), *glomus intraradices* (FJ648425) (Lee and Young 2009), *rhizopus oryzae* (CM022358) (Nguyen et al. 2020), and *apiotrichum gamsii* (MZ439919) (Li et al. 2023).

The average depth of the coverage-depth map was 6398.43× (Supplementary Figure 1), and the mitochondrial genome was 43,837 bp long with a GC content of 24.93%. The structures of genes containing introns were shown in Supplementary Figure 2. The mitochondrial genome of *R. microsporus* is composed of 37.62% adenine, 13.06% guanine, 37.45% thymine, and 11.87% cytosine. Analysis of the *R. microsporus* mitochondrial genome revealed 24 open-reading frames, which included 14 core PCGs (*cox1, cox2, cox3, atp6, atp8, atp9, cob, nad1, nad2, nad3, nad4, nad4L, nad5,* and *nad6*), 3 free-standing ORFs, and 7 intronic ORFs (Figure 2). Notably, the proteins encoded by the free-standing ORFs had unknown functions. The *R. microsporus* mitochondrial genome was found to contain 11 introns, with 8 belonging to Group IB, 3 to Group IA, and 1 to Group I(derived). Intronic ORFs encoding LAGLIDADG homing endonucleases or GIY-YIG homing endonucleases were present in some of the introns. The mitochondrial genome of *R. microsporus* was found to contain two ribosomal RNA genes, the small subunit (*rns*) and the large subunit (*rnl*), as well as 24 transfer RNA genes. Phylogenetic analysis demonstrated that *R. microsporus* is a sister species to *Rhizopus* oryzae, as depicted in Figure 3.

## 4. Discussion and conclusion

By utilizing the mitochondrial genome, we can gain a more comprehensive comprehension of the phylogenetic relationship between species (Zhang et al. 2020; Ren et al. 2021; Zhang et al. 2022, 2023; Gao et al., 2024). The absence of a mitochondrial reference genome for Rhizopodaceae, particularly *Rhizopus* species, impedes the application of mitochondrial genome for classifying and investigating the phylogenetic relationship of early-diverging fungi (Caramalho et al. 2019). In this research, we acquired a full mitochondrial genome of *Rhizopus* species. It was 43,837 bp in length, with a GC content of 24.93%. This genome included 14 core protein-coding genes (PCGs), 3 independent ORFs, 7 intronic ORFs, 24 tRNAs, and 2 rRNA genes. The *R. microsporus* mitogenome is the smallest among the three mitogenomes in the *Rhizopus* genus, 33.94% smaller than *R. oryzae* and 23.62% smaller than *R. arrhizus*, respectively, indicating that the *R. microsporus* mitochondrial genome has undergone contraction during the evolutionary process. By employing the BI phylogenetic inference method, we were able to construct phylogenetic trees for 25 early differentiation fungi, with strong support for major clades; this demonstrated that *R. microsporus* is most closely related to *Rhizopus oryzae*. This study provides us with valuable information that is indispensable for the distinction and recognition of *Rhizopus* species, thus increasing our understanding of mitochondrial evolution and the varieties of early-emerging fungi.

## Supplementary Material

Supplemental Material

## Data Availability

The genome sequence data that support the findings of this study are openly available in GenBank of NCBI at https://www.ncbi.nlm.nih.gov/ under the accession no. OR664025. The associated BioProject, SRA, and Bio-Sample numbers are PRJNA1025866, SRR26320846 and SAMN37723663, respectively.
