# Supplementary material for: Mitochondrial genomic characteristics and phylogenetic analysis of a brewing fungus, Rhizopus microsporus Tiegh. 1875 (Mucorales: Rhizopodaceae)
Source: Mitochondrial DNA B Resour. 2024 May 20;9(5):657–62. doi: 10.1080/23802359.2024.2356133 (PMC11107855; doi:10.1080/23802359.2024.2356133)
Supplement: Supplemental Material [file TMDN_A_2356133_SM5524.docx]

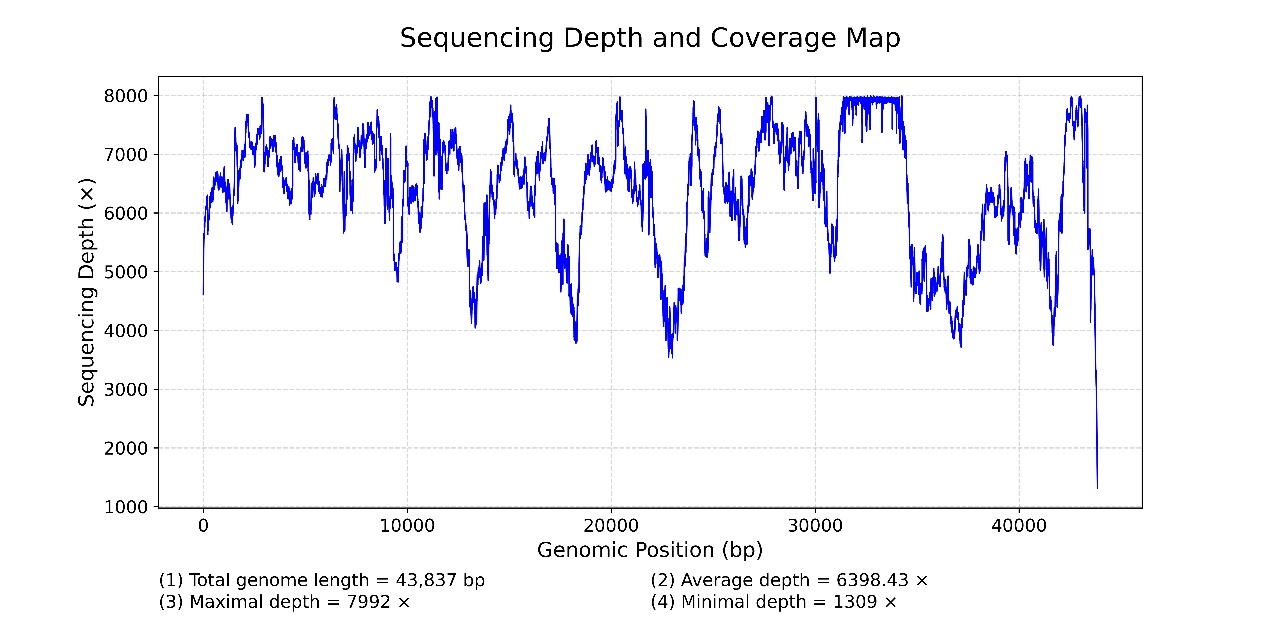


**Figure S1** Sequencing depth and coverage map of *Rhizopus microspores* mitochondrial genome.


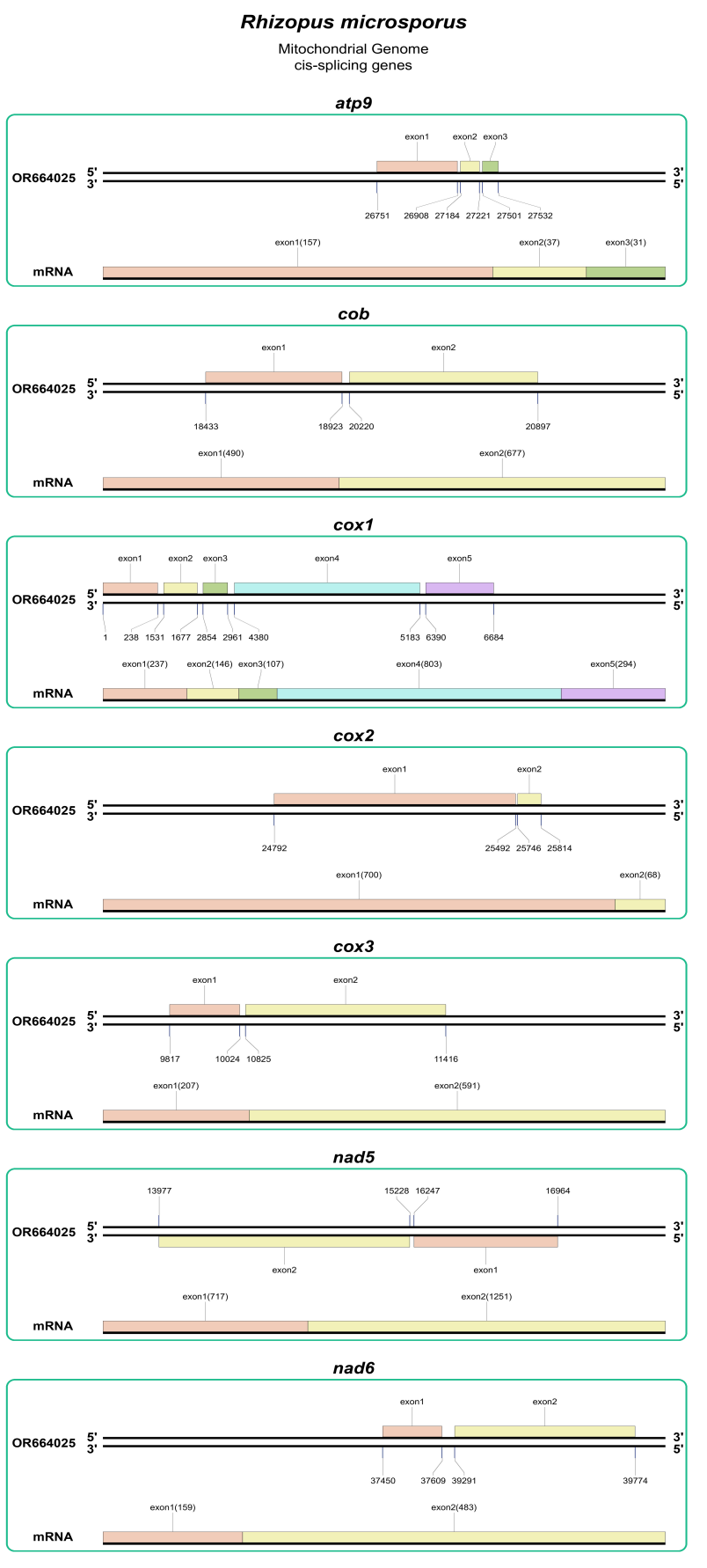


**Supplementary Figure S2** Cis-splicing genes of the *Rhizopus microspores* mitochondrial genome.
